# Supplementary material for: Evidence supported by Mendelian randomization: impact on inflammatory factors in knee osteoarthritis
Source: Front Med (Lausanne). 2024 May 28;11:1382836. doi: 10.3389/fmed.2024.1382836 (PMC11165061; doi:10.3389/fmed.2024.1382836)
Supplement: Supplementary file 1 [file Data_Sheet_1.ZIP › data/GCST90274822_5e6/822.leaveoneout.pdf]

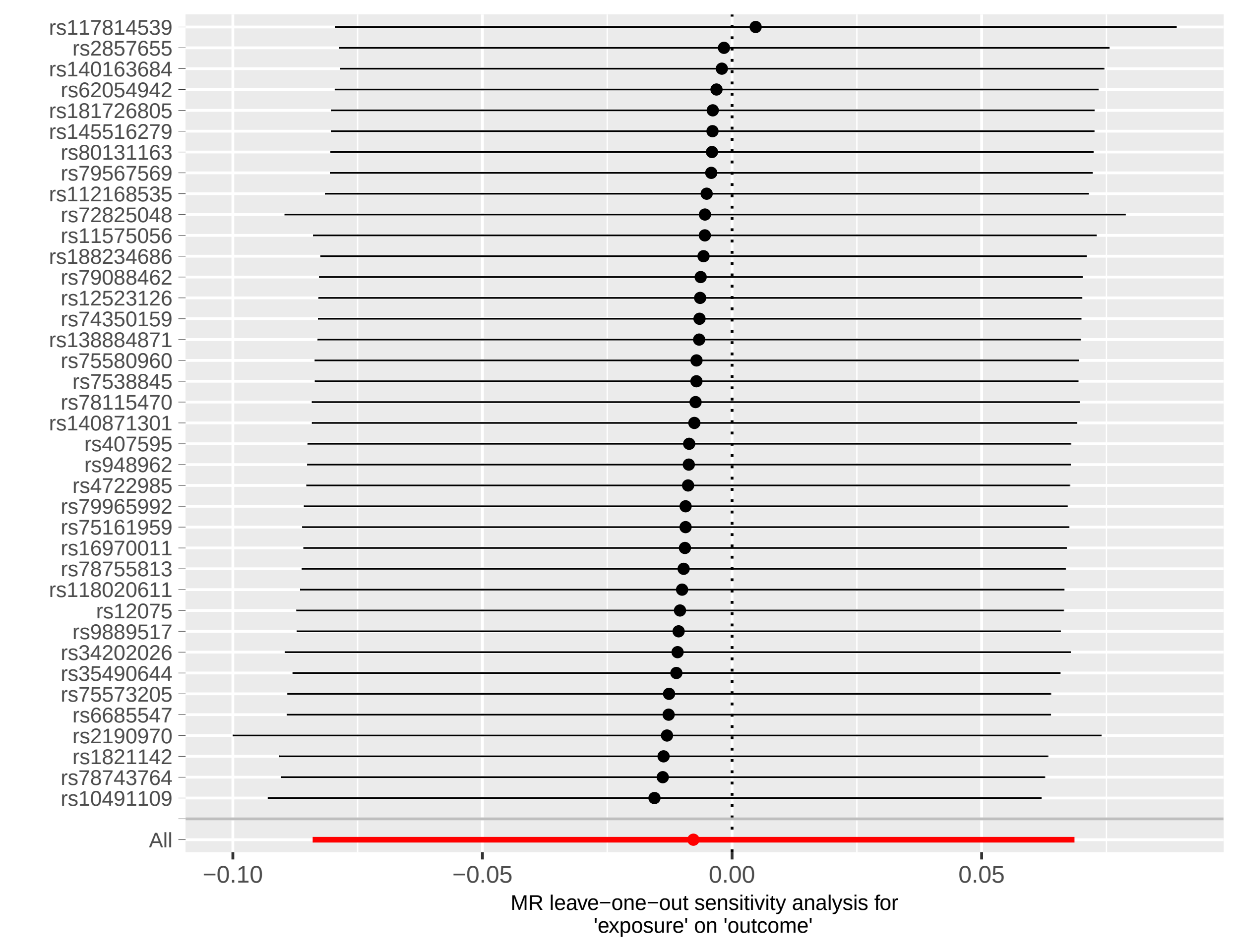
rs117814539
rs2857655
rs140163684
rs62054942
rs181726805
rs145516279
rs80131163
rs79567569
rs112168535
rs72825048
rs11575056
rs188234686
rs79088462
rs12523126
rs74350159
rs138884871
rs75580960
rs7538845
rs78115470
rs140871301
rs407595
rs948962
rs4722985
rs79965992
rs75161959
rs16970011
rs78755813
rs118020611
rs12075
rs9889517
rs34202026
rs35490644
rs75573205
rs6685547
rs2190970
rs1821142
rs78743764
rs10491109
All
−0.10
−0.05
0.00
0.05
MR leave−one−out sensitivity analysis for
'exposure' on 'outcome'
